# Expression and prognostic value of cell-cycle-associated genes in gastric adenocarcinoma

**DOI:** 10.1186/s12876-018-0811-1

**Published:** 2018-06-08

**Authors:** Dongya Wang, Haige Zhu, Meng Guo, Xiaotong Fan, Shuangshuang Hu, Kemin Yan, Jia Sun, Jiaojiao Wang, Miaomiao Li, Haijuan Xiao, Zhiguo Liu

**Affiliations:** 1 General Surgery Department, The Affiliated Hospital of Jinggangshan University, Ji’an, 343000 Jiangxi China; 2 School of Life Science, Fudan University, Shanghai, 200032 China; 3 Xijing Hospital of Digestive Diseases, Fourth Military Medical University, Xi’an, 710000 Shaanxi China; 4 State Key Laboratory of Cancer Biology, Xijing Hospital of Digestive Diseases, Fourth Military Medical University, No. 127, West Changle Road, Xincheng District, Xian, Xi’an, 710032 Shaanxi China; 5 Department of Oncology, Affiliated Hospital of Shaanxi University of Chinese Medicine, Xianyang, 712000 People’s Republic of China

**Keywords:** Gastric adenocarcinoma, Cell cycle, Gene expression, Tumor stages, Prognostic implications

## Abstract

**Background:**

Gastric carcinoma is a malignant disease, and gastric adenocarcinoma (GAC) is the most common histological type. Molecular profiling of GAC has been extensively performed, but few have focused on the clinical significance of gene clusters of the cell cycle.

**Methods:**

We investigated the genetic profile of cell-cycle-associated genes in a GAC cohort. The mRNA expression and clinical data were downloaded from TCGA, according to cBioportal. We conducted a series of analyses to detect the relationships between these genes and GAC.

**Results:**

From all the patients, 5 clusters were identified based on mRNA expression of 122 cell-cycle-associated genes. Cluster 1 showed the worst prognosis and is characterized by extremely high expression of *WEE2* and *CCNE1*. Comparison of the gene patterns showed that 16 genes expressed were distinctly varied between each cluster. In addition, investigations into the prognostic role of the 16 genes suggested that high expression of *ESPL1* and *MCM5* were significantly correlated with favorable outcomes. Moreover, we detected that *ESPL1* and *MCM5* gene expression were negatively correlated with GAC pathologic stage progression.

**Conclusions:**

This study revealed a gene expression pattern of the cell cycle in different GAC subgroups, and suggested individual genes were associated with the clinical outcome and AJCC stages. These results suggest a novel prognostic strategy for GAC and provide information for patient stratification and trials of targeted therapies.

**Electronic supplementary material:**

The online version of this article (10.1186/s12876-018-0811-1) contains supplementary material, which is available to authorized users.

## Background

Gastric carcinoma (GC) remains the fifth most prevalent cancer and the third leading cause of cancer-related deaths worldwide [1, 2]. Gastric adenocarcinoma (GAC) is the main type of GC and is associated with poor survival rates [1]. Although the incidence of GAC has been reduced over the past years, it imposes a critical issue globally [3]. The characteristics of diverse histological (phenotypes) and genotypes manifest that GAC is a heterogeneous disease [4]. Previous studies uncovered the genetic profiling of GAC by performing gene expression or DNA sequencing, but rare profiling led to alterations of gene panels associated with specific biological events [5–7]. The cell cycle process is a highly organized event and regulated duplication of genetic material and cell division, and aberrant cell cycle activity is a hallmark of cancer [8]. A comprehensive molecular characterization study elicited frequent amplifications of cell cycle mediators (CCNE1, CCND1 and CDK6) in GAC; however, it remains unclear if specific patterns of cell-cycle-associated gene expression across different subtypes or stages bears any significance on patient outcome or is correlated with genetic alterations [9].

Tumor stages are generally used to characterize disease progression and determine metastasis and prognosis [10]. Gene expression involved in the development of GC is also variant in different stages [11]. In this study, we focused on mRNA expressing variations of genes associated with the cell cycle in different GAC subgroups, as well as its correlation to tumor stages. We identified significantly differentially expressed genes in GAC samples and the prognostic impact [12]. Specific genes that impacted the outcomes showed a variant expression panel between different stages. Finally, we report a unique set of cell-cycle-associated genes in GAC that serve as a divider of biological characteristics, which revealed the potential for therapeutic strategy targeting cell-cycle-associated genes.

## Methods

### Samples and database

We obtained RNA-Seq data and the corresponding clinical records of 415 gastric adenocarcinoma patients (GAC) of TCGA from cBioPortal for Cancer Genomics (http://cbioportal.org) [13]. We filtrated the data base on whether the mRNA z-score, tumor stage and overall survival were clearly recorded. Collectively, the data set included 228 samples for the clustering study and 206 samples for the stage study, respectively.

A panel of cell-cycle-associated genes was derived from the KEGG pathway database (http://www.kegg.jp/kegg/), as previously described [14]. In total, 124 genes were listed; however, mRNA expression data of genes *MYC* and *CCND1* were unobtainable, and the remaining 122 candidate genes were analyzed (Additional file 1: Table S1).

### Bioinformatics

Hierarchical cluster analysis of 122 genes expressed in each sample was used to group samples with similar gene expression patterns. Samples with differential cell-cycle-associated gene expression between separate clusters were identified from the whole data set. The expression levels of GAC patients were shown as mRNA z-scores and grouped using the hierarchical clustering algorithm in the Gene Cluster 3.0 program [15]. The heat-map of cluster indication and tumor stage divided pattern was generated using the Java Treeview program [16].

### Prognostic implication analyses

To evaluate the relationship between the cell-cycle-associated genes and patient clinical outcome, we used GraphPad Prism 6 for Windows (GraphPad Software, Inc., California, US; Version 6.01, 2012) for construction and overall survival (OS) comparisons in different gastric adenocarcinoma clusters and different tumor stages. Additionally, OS difference analysis between low and high expression cohorts were conducted in GraphPad Prism 6.

### Statistical analysis

Survival curves were plotted using a Kapla-Meier analysis and compared using the log-rank test in GraphPad Prism 6. Associations between clinical characteristics and clustering variable were analyzed by Fisher’s exact test and Pearson/Spearman correlation. Difference of gene expression between clusters was conducted by ANOVA analysis. Correlation between each element was conducted by regression analysis. Both results were performed in SPSS 19.0 (IBM, Inc., New York, US). A *P* value of less than 0.05 was considered statistically significant.

## Results

### Expression profile of cell-cycle-associated genes in GAC

To understand biological functions and utilities of the biological system, we retrieved 124 cell-cycle-associated genes (PATHWAY: map04110) from the KEGG database. Additionally, we selected 122 genes that were detectable and sorted the mRNA expression values from the RNA-seq data of GAC (Additional file 2: Figure S1).

**Fig. 1:**
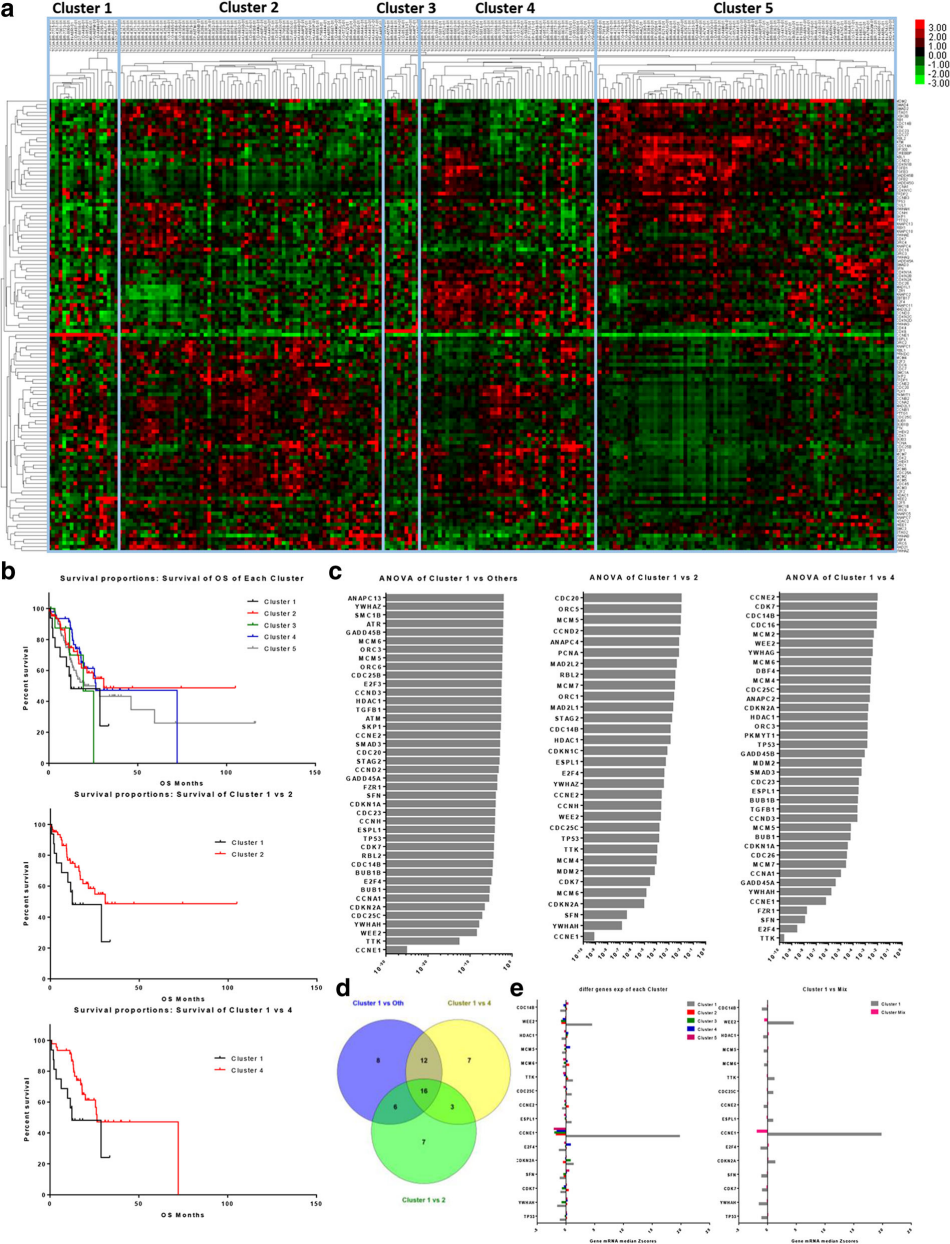
Differential expression of cell-cycle-associated genes in each cluster. a, The GAC patients were divided into 5 clusters based on mRNA expression levels. **b**, Survival difference between all clusters (top chart), cluster 1 versus cluster 2 (middle chart) and cluster 1 versus cluster 4. **c**, Differentiation of gene expression between clusters were detected: cluster 1 and other clusters (left); cluster 1 and cluster 2 (middle); cluster 1 and cluster 4 (right). **d**, There are 16 genes presented in the overlap of differentiation sets. **e**, *WEE2* and *CCNE1* showed the most distinct changes in comparison, no matter of cluster 1 and other clusters, nor of cluster 1 and other total patients

The expression levels of the 122 genes were calculated by mRNA z-scores compared to the expression distribution of each gene from tumors that were diploid for the genes in 415 GAC cases (RNA Seq V2 RSEM), based on TCGA data in GC. Following the clinical filtrated criteria, we achieved 228 cases with survival records to perform cluster analysis. Five clusters of GAC were distinguished based on the gene expression panels (Fig. 1a). The statistical analysis of the clinical and histological characteristics of each clusters was performed, and the result shows that the nodes pathologic stages and tumor stages was significantly different (The characteristic of histological types was excluded for the reason of the incompleteness of data) (Table 1). Moreover, correlational analysis showed the significant correlations of the three parameters and clustering situation (Table 1).

**Table 1:** The clinical and histological characteristics of each clusters

Cluster		1	2	3	4	5	*P*-value	Correlation coefficient
		(*n* = 15)	(*n* = 10)	(*n* = 47)	(*n* = 63)	(*n* = 71)		
Age (mean)		62.7	64.1	67.2	65.2	63.6	0.406	−0.061
Gender	Male	12	7	25	42	40	0.255	0.178^c^
	Female	3	3	22	21	31		
Race	Asian	5	3	12	9	15	0.309	0.093
	White	10	7	30	47	49		
	Black	0	0	3	1	0		
	NA^a^	0	0	2	6	7		
Grade	G1	0	0	0	0	3	0.104	0.178^c^
	G2	6	2	14	12	6		
	G3	9	8	32	50	60		
	GX	0	0	1	1	2		
Histological diagnosis (GAC)	Signet ring type	1	0	2	2	6	/	/
	Diffuse type	2	2	10	18	34		
	NOS^b^	12	8	35	43	31		
History of other malignancy	Yes	1	0	1	1	4	0.595	−.057
	No	14	10	46	62	67		
Neoadjuvant therapy	Yes	0	0	0	0	0	/	/
	No	15	10	47	63	71		
Tumor pathologic (AJCC PT)	T1	1	0	1	3	1	0.136	.089
	T2	3	1	13	16	11		
	T3	8	5	15	34	30		
	T4	3	4	18	10	29		
Nodes pathologic (AJCC PN)	N0	6	3	25	24	17	0.009	0.165^c^
	N1	4	4	7	17	20		
	N2	1	2	8	7	15		
	N3	3	0	7	15	19		
	NX	1	1	0	0	0		
Metastasis pathologic (AJCC PM)	M0	14	10	43	56	61	0.211	0.093
	M1	0	0	0	6	7		
	MX	1	0	4	1	3		
Tumor stage	Stage I	1	0	10	11	5	0.006	0.252^d^
	Stage II	9	8	20	25	20		
	Stage III	4	2	17	20	38		
	Stage IV	1	0	0	7	8		

^a^NA represents not available

^b^NOS represents not otherwise specified

^c^The correlation was significant when the confidence coefficient (double measurement) was 0.05

^d^The correlation was significant when the confidence coefficient (double measurement) was 0.01

We compared the median survival and survival curves between each cluster further and the results showed that cluster 1 demonstrated the worst prognosis (12.35 months) compared to other clusters (26.31 months) (Fig. 1b). In contrast, clusters 2 and 4 showed favorable prognosis with a median survival of 30.88 months and 26.45 months, respectively (Table 2). Comparison of survival curves revealed a significant difference between outcomes of cluster 1 and cluster 4 (*P* = 0.0278) (Additional file 1: Table S2).

**Table 2:** Median survival of each cluster

	Cluster 1	Cluster 2	Cluster 3	Cluster 4	Cluster 5
Number of rows	19	71	10	47	81
Median survival (Months)	12.35	30.88	19.32	26.45	19.94

### Variation of cell-cycle-associated gene expression in different clusters

Additionally, we examined the genes that expressed variation between each cluster. According to the filter criteria of significant difference (*P* < 0.01), there were 42 differentially expressed genes of cluster 1 versus the other clusters, 32 differentially expressed genes of cluster 1 versus cluster 2 and 38 differentially expressed genes of cluster 1 versus cluster 4 (Fig. 1c). A cross-reference of the three gene groups showed that 16 genes were included in the overlaps (Fig. 1d). A comparison of the 16 genes of mRNA expression demonstrated that *WEE2* and *CCNE1* were dramatically elevated in cluster 1 (*P* = 2.89085E-09 and *P* = 6.96046E-26, respectively) (Fig. 1e and Additional file 1: Table S3).

### Expression of cell-cycle-associated genes were correlated with prognosis in GAC

**Fig. 2:**
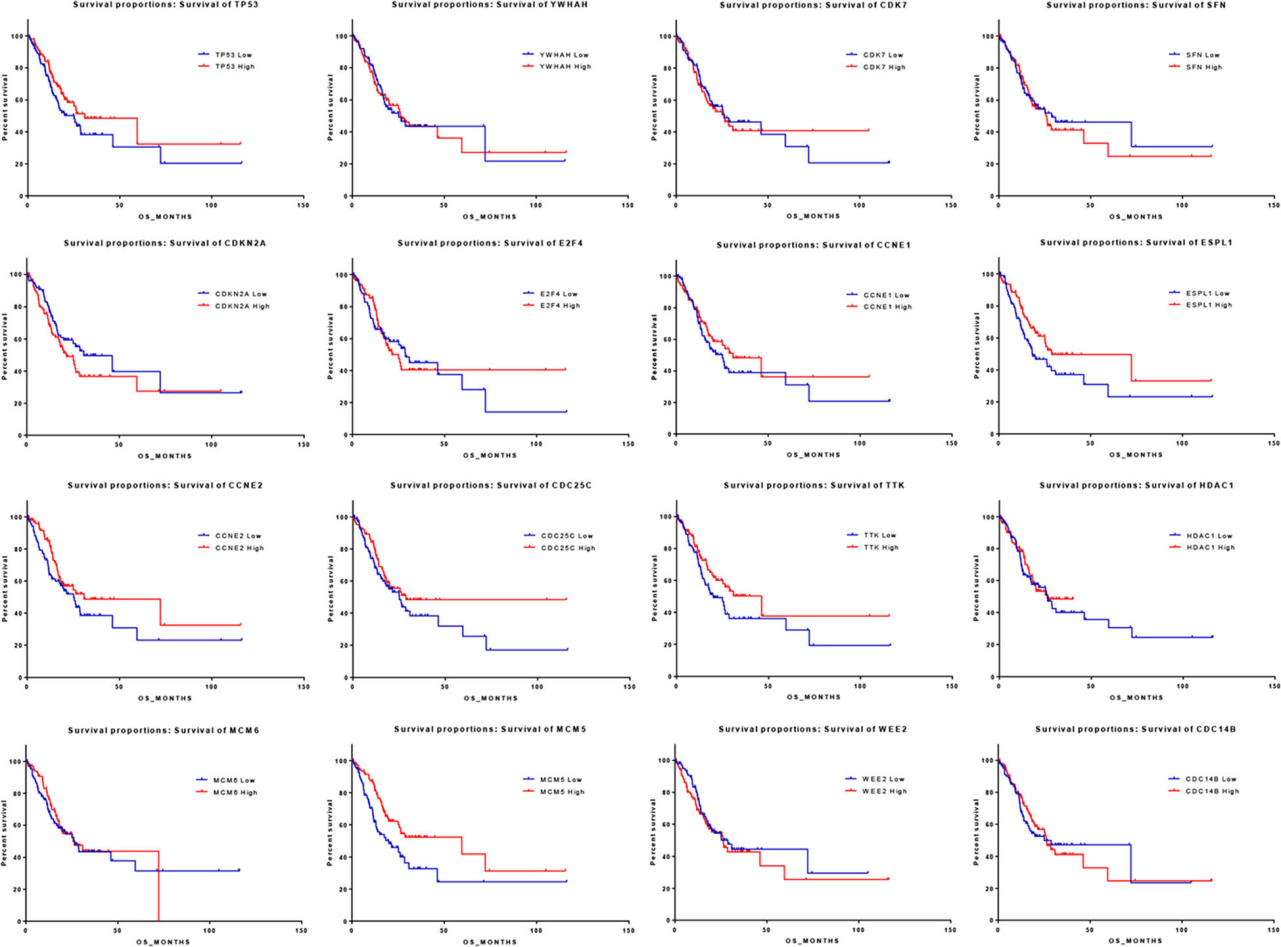
Survival curves represented the prognostic impact of 16 genes. The cases were equally divided into low-expression and high-expression, according to the mRNA expression levels. High-expression of *ESPL1* and *MCM5* indicated a favorable overall survival

To address the prognostic roles of genes with cluster-differential expression, we evenly divided the samples into two groups based on the mRNA z-scores from high values to low values. Survival curves according to each single gene are shown in Fig. 2. Comparison of the median survival and survival curves in the 16 genes between low-expression and high-expression groups revealed that *ESPL1* and *MCM5* were significantly associated with clinical outcome (*P* = 0.0448 and 0.0048, respectively) (Table 3). High expression of *ESPL1* and *MCM5* indicated a favorable median survival (18.33 vs 28.71 months and 19.94 vs 59.49 months, respectively). In addition, elevated *CCNE2* and *TTK* expression also showed a trend of correlation with good prognosis, but with a non-significant difference (Table 3).

**Table 3:** Comparison of survival curves of low and high expression groups of each cluster differ-expressed gene by Log-rank (Mantel-Cox) test

Cluster differ-expressed genes	Chi square	df	P value	Significant different^a^	Median survival of Low-Exp. (Months)	Median survival of High-Exp. (Months)
*TP53*	2.283	1	0.1308	ns	25.16	30.88
*YWHAH*	0.02811	1	0.8669	ns	25.16	26.31
*CDK7*	0.1239	1	0.7248	ns	26.08	26.31
*SFN*	0.06809	1	0.7941	ns	28.71	26.08
*CDKN2A*	2.168	1	0.141	ns	30.88	21.42
*E2F4*	0.04874	1	0.8253	ns	28.71	25.03
*CCNE1*	1.126	1	0.2887	ns	25.16	30.88
*ESPL1*	4.025	1	0.0448	*	18.33	28.71
*CCNE2*	3.125	1	0.0771	ns	25.16	30.88
*CDC25C*	1.725	1	0.189	ns	25.16	28.71
*TTK*	3.175	1	0.0748	ns	19.88	46.22
*MCM6*	0.2145	1	0.6433	ns	26.45	25.59
*MCM5*	7.939	1	0.0048	**	19.94	59.49
*HDAC1*	0.3977	1	0.5283	ns	26.08	26.45
*WEE2*	0.6097	1	0.4349	ns	28.71	26.31
*CDC14B*	0.1163	1	0.7331	ns	25.03	26.31

^a^ns represents non-significance

*P values were less than 0.05 and equal or greater than 0.01

**P values were less than 0.01

### Expression of *ESPL1* and *MCM5* were associated with tumor stage progression

**Fig. 3:**
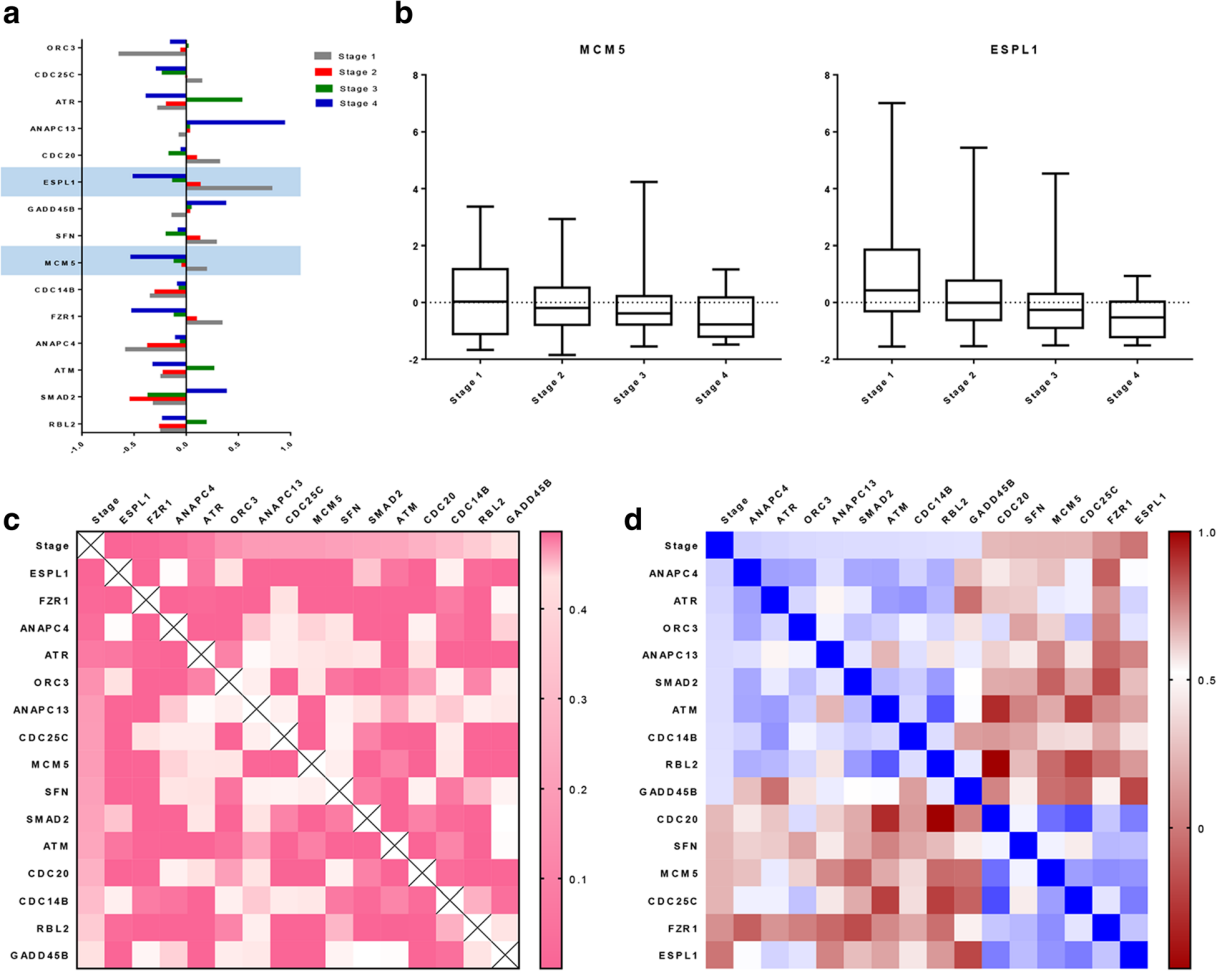
Expression of ESPL1 and MCM5 was decreased correlating to tumor stage progression. **a**, Expression of cluster-differential genes were divided by AJCC stages, and *ESPL1* and *MCM5* were significantly changed. **b**, *ESPL1* and *MCM5* were negatively correlated with tumor stage progression. **c**, Significance of the correlation in stage versus 15 genes and between each gene were demonstrated in the heat map (“Red” represents smaller *P* values and “White” represents lager P values). **d**, Pearson correlation of stage versus 15 genes and between each gene were demonstrated in the heat map (“Dark red” represents negative correlation and “Blue” represents positive correlation)

To detect the impacted factors of differential gene expression, we filtered out a sample set with intact pathologic records. According to investigating the expression of cluster 1-specific genes corresponding to each clinical manifestation, we detected obvious differences of the 16 genes expressed in each tumor stage (Fig. 3a). In addition, *ESPL1* and *MCM5* expression were negatively correlated with tumor stage progression (Fig. 3b, c), and regression analysis indicated that *ESPL1* and *MCM5* were correlated to tumor stages (Pearson correlation = − 0.25713; *P* = 9.54E-05 and Pearson correlation = − 0.13982; *P* = 0.023, respectively) (Additional file 1: Table S4).

Additionally, we investigated the correlation between cluster 1 specifically expressed genes and tumor stages. There were 15 genes, including *ESPL1* and *MCM5*, that showed relativity (*P* < 0.05) with tumor stage changing (Fig. 3b) (Additional file 1: Table S4). A correlation matrix of each gene and stage showed that *FZR1* was also weakly correlated with stages (Pearson correlation = − 0.20806; *P* = 0.001). What’s more, we detected that the expression of *ESPL1* was positively correlated with the expression of *FZR1, SFN, MCM5, CDC20* and *CDC25C* and negatively correlated with the expression of *GADD45B* and *ANAPC13. MCM5* was positively correlated with the expression of *CDC25C*, *ESPL1*, *FZR1* and *CDC20* and negatively correlated with the expression of *SMAD2*, *RBL2*, *GADD45B* and *ANAPC13* (Table 4) (Fig. 3c, d)*.*

**Table 4:** Correlation between *MCM5*/*ESPL1* and tumor stages-mutative genes

Correlation	*MCM5*			*ESPL1*		
	Genes	Person correlation ^†^	Significance^‡^	Genes	Person correlation^†^	Significance^‡^
Positive	*CDC25C*	0.379754	9.02914E-09	*FZR1*	0.231261	0.000412193
	*ESPL1*	0.419389	1.75479E-10	*SFN*	0.259065	8.49402E-05
	*FZR1*	0.442699	1.34359E-11	*MCM5*	0.419389	1.75479E-10
	*CDC20*	0.561659	7.9853E-19	*CDC20*	0.502711	6.81739E-15
				*CDC25C*	0.51131	2.0258E-15
Negative	*SMAD2*	−0.29366	9.18019E-06	*GADD45B*	−0.34282	2.27821E-07
	*RBL2*	−0.27645	2.88184E-05	*ANAPC13*	−0.22992	0.000442825
	*GADD45B*	−0.26908	4.59871E-05			
	*ANAPC13*	−0.2219	0.000673931			

^†^Person correlation coefficient of paired genes

^‡^Significance of regression analysis was represented by P value

In summary, we studied the expression profiles of 122 cell-cycle-associated genes and sorted the GAC samples into 5 clusters according to mRNA z-score distribution. Sixteen specific genes showed differential expression between each cluster that were identified. We examined the prognostic roles of the 16 genes and detected that *ESPL1* and *MCM5* were significantly associated with overall survival. Moreover, we detected that the expression of *ESPL1* and *MCM5* were negatively correlated with tumor stage progression. These results suggested the significance of cell-cycle-related gene expression in the development and progression of GAC and provided potential targets for GAC therapy.

## Discussion

Uncontrolled tumor cell proliferation via aberrant expression of various cell cycle genes is one of the most essential features in multiple cancers. Therefore, cell-cycle-regulated genes are considerable targets in cancer therapy [17]. Through this study of the mRNA expression of 122 cell-cycle-associated genes, we demonstrated a molecular profile, which defined five genomic clusters of GAC. Among them, cluster 1 manifested the worst survival and was characterized by dramatically elevated expression of *WEE2* and *CCNE1*. *WEE2* is an oocyte-specific protein tyrosine kinase that phosphorylates and inhibits CDK1, and acts as a key regulator of meiosis during both prophase I and metaphase II [18]. In GAC, *WEE2* might play a similar role by mediated the CDK1 phosphorylation. Amplification of *CCNE1* (G1/S-specific cyclin-E1) is associated with poor outcome in breast, lung, and other solid cancers [19]. However, prognosis implication analysis of single genes did not show any correlation of unfavorable outcome and highly expressed *WEE2* and *CCNE1* (*P* = 0.4349 and *P* = 0.2887, respectively). This discrepancy revealed that *WEE2* and *CCNE1* were indirectly regulated the GAC by triggering other molecular events.

In entire GAC cases, we detected that elevated *ESPL1* and *MCM5* expression were significantly associated favorable prognosis, and the expression variability were reversely consisted with stage progression. *ESPL1*, a protease (Separase) encoded gene, was reported overexpressing in mammary adenocarcinomas and related to tumor initiation and progression, but not mentioned in GAC [20]. According to cleavage of cohesin complex subunit, *ESPL1* might promote metaphase/anaphase transition during the cell cycle in GAC [21]. *MCM5*, minichromosome maintenance complex component 5, which encoded protein is involved in the initiation of DNA replication and the effect might reduce the accumulation of genetic variation in GAC. The prognostic analysis of *MCM5* in current study showed that the upregulated *MCM5* was associated with a favorable prognosis. This result was in accordance with an previous immunohistochemical study, which suggested that patients with high MCM5 expression had significantly shorter survival times and MCM5 was associated with clinicopathological parameters in gastric adenocarcinoma [22]. Besides, additional 13 genes specifically expressed in cluster 1 were also correlated to tumor stage. The regression analysis showed that *ESPL1* and *MCM5* was positively correlated (Person = 0.419389, *P* = 1.75479E-10) and both them were correlated to other cell cycle-specific genes expression, suggesting a co-regulation effect through genes expression changing in GAC.

Although previous studies revealed the significant role of cell cycle associated genes in gastric cancer, most works were focus on individual genes [23–25]. In current study, we discuss the panorama of cell cycle associated genes in GAC samples. Those results might appeal to the further studies of various cell cycle inhibitors with therapeutic potential. For example, evaluation of cell cycle derangement in thyroid tumors may serve as a useful tool for both DTC diagnosis and prognosis [26]; PLK1 and AK inhibitors display the potential for being employed in innovative therapeutic strategies for improving T-ALL patient outcome [27]; Inhibition of Aurora and Polo-like kinases suggest that targeting G2-M regulators may represent a novel approach for treatment of human [28].

## Conclusions

Irrespective of tissue origin and adjuvant therapy, this work revealed the gene expression profile of cell cycle association in GAC. The impact and functions of distinctive genes need to be further investigated. We believe these results will facilitate the exploration of novel therapies, ultimately improving clinical outcome from this intractable disease.

## Additional files

Additional file 1: **Table S1.** Gene listed in KEGG cell cycle pathway (hsa04110). **Table S2.** Comparison of Survival Curves of each cluster by Log-rank (Mantel-Cox) test. **Table S3.** Comparison of individual gene expressions of each cluster. **Table S4.** Correlation between Cluster-specific genes expression and tumor stages. (DOCX 24 kb).

Additional file 2: **Figure S1.** Networks of cell cycle associated genes. The interaction values between each connected gene were exported from String-db. (TIF 1159 kb).

## Abbreviations

AJCC: American Joint Committee on Cancer
DTC: Differentiated thyroid carcinoma
GAC: Gastric adenocarcinoma
GC: Gastric carcinoma
KEGG: Kyoto encyclopedia of genes and genomes
RNA: Ribonucleic acid
RNA-seq: Whole transcriptome sequencing
T-ALL: T-cell acute lymphoblastic leukemia
TCGA: The cancer genome atlas
