# Supplementary material for: Expression and prognostic value of cell-cycle-associated genes in gastric adenocarcinoma
Source: BMC Gastroenterol. 2018 Jun 8;18:81. doi: 10.1186/s12876-018-0811-1 (PMC5994033; doi:10.1186/s12876-018-0811-1)
Supplement: Supplementary file 1 — Table S1. Gene listed in KEGG cell cycle pathway (hsa04110). Table S2. Comparison of Survival Curves of each cluster by Log-rank (Mantel-Cox) test. Table S3. Comparison of individual gene expressions of each cluster. Table S4. Correlation between Cluster-specific genes expression and tumor stages. (DOCX 24 kb). [file 12876_2018_811_MOESM1_ESM.docx]

Additional file 1 Table S1: Gene listed in KEGG cell cycle pathway (hsa04110)

| Gene Code | Gene Name | Gene Annotation | KEGG ORTHOLOGY Code |
| --- | --- | --- | --- |
| 595 | CCND1 | cyclin D1 | KO:K04503 |
| 894 | CCND2 | cyclin D2 | KO:K10151 |
| 896 | CCND3 | cyclin D3 | KO:K10152 |
| 1019 | CDK4 | cyclin dependent kinase 4 | KO:K02089 |
| 1021 | CDK6 | cyclin dependent kinase 6 | KO:K02091 |
| 5925 | RB1 | RB transcriptional corepressor 1 | KO:K06618 |
| 5933 | RBL1 | RB transcriptional corepressor like 1 | KO:K04681 |
| 5934 | RBL2 | RB transcriptional corepressor like 2 | KO:K16332 |
| 25 | ABL1 | ABL proto-oncogene 1, non-receptor tyrosine kinase | KO:K06619 |
| 3065 | HDAC1 | histone deacetylase 1 | KO:K06067 |
| 3066 | HDAC2 | histone deacetylase 2 | KO:K06067 |
| 1869 | E2F1 | E2F transcription factor 1 | KO:K17454 |
| 1870 | E2F2 | E2F transcription factor 2 | KO:K09389 |
| 1871 | E2F3 | E2F transcription factor 3 | KO:K06620 |
| 1874 | E2F4 | E2F transcription factor 4 | KO:K04682 |
| 1875 | E2F5 | E2F transcription factor 5 | KO:K04682 |
| 7027 | TFDP1 | transcription factor Dp-1 | KO:K04683 |
| 7029 | TFDP2 | transcription factor Dp-2 | KO:K09392 |
| 2932 | GSK3B | glycogen synthase kinase 3 beta | KO:K03083 |
| 7040 | TGFB1 | transforming growth factor beta 1 | KO:K13375 |
| 7042 | TGFB2 | transforming growth factor beta 2 | KO:K13376 |
| 7043 | TGFB3 | transforming growth factor beta 3 | KO:K13377 |
| 4087 | SMAD2 | SMAD family member 2 | KO:K04500 |
| 4088 | SMAD3 | SMAD family member 3 | KO:K04500 |
| 4089 | SMAD4 | SMAD family member 4 | KO:K04501 |
| 4609 | MYC | MYC proto-oncogene, bHLH transcription factor | KO:K04377 |
| 7709 | ZBTB17 | zinc finger and BTB domain containing 17 | KO:K10500 |
| 1029 | CDKN2A | cyclin dependent kinase inhibitor 2A | KO:K06621 |
| 1030 | CDKN2B | cyclin dependent kinase inhibitor 2B | KO:K04685 |
| 1031 | CDKN2C | cyclin dependent kinase inhibitor 2C | KO:K06622 |
| 1032 | CDKN2D | cyclin dependent kinase inhibitor 2D | KO:K06623 |
| 1027 | CDKN1B | cyclin dependent kinase inhibitor 1B | KO:K06624 |
| 1028 | CDKN1C | cyclin dependent kinase inhibitor 1C | KO:K09993 |
| 1026 | CDKN1A | cyclin dependent kinase inhibitor 1A | KO:K06625 |
| 898 | CCNE1 | cyclin E1 | KO:K06626 |
| 9134 | CCNE2 | cyclin E2 | KO:K06626 |
| 1017 | CDK2 | cyclin dependent kinase 2 | KO:K02206 |
| 6500 | SKP1 | S-phase kinase associated protein 1 | KO:K03094 |
| 8454 | CUL1 | cullin 1 | KO:K03347 |
| 9978 | RBX1 | ring-box 1 | KO:K03868 |
| 6502 | SKP2 | S-phase kinase associated protein 2 | KO:K03875 |
| 890 | CCNA2 | cyclin A2 | KO:K06627 |
| 8900 | CCNA1 | cyclin A1 | KO:K06627 |
| 990 | CDC6 | cell division cycle 6 | KO:K02213 |
| 8318 | CDC45 | cell division cycle 45 | KO:K06628 |
| 8317 | CDC7 | cell division cycle 7 | KO:K02214 |
| 10926 | DBF4 | DBF4 zinc finger | KO:K06629 |
| 983 | CDK1 | cyclin dependent kinase 1 | KO:K02087 |
| 891 | CCNB1 | cyclin B1 | KO:K05868 |
| 9133 | CCNB2 | cyclin B2 | KO:K21770 |
| 85417 | CCNB3 | cyclin B3 | KO:K21771 |
| 994 | CDC25B | cell division cycle 25B | KO:K05866 |
| 995 | CDC25C | cell division cycle 25C | KO:K05867 |
| 7534 | YWHAZ | tyrosine 3-monooxygenase/tryptophan 5-monooxygenase activation protein zeta | KO:K16197 |
| 7529 | YWHAB | tyrosine 3-monooxygenase/tryptophan 5-monooxygenase activation protein beta | KO:K16197 |
| 10971 | YWHAQ | tyrosine 3-monooxygenase/tryptophan 5-monooxygenase activation protein theta | KO:K16197 |
| 7531 | YWHAE | tyrosine 3-monooxygenase/tryptophan 5-monooxygenase activation protein epsilon | KO:K06630 |
| 7533 | YWHAH | tyrosine 3-monooxygenase/tryptophan 5-monooxygenase activation protein eta | KO:K16198 |
| 7532 | YWHAG | tyrosine 3-monooxygenase/tryptophan 5-monooxygenase activation protein gamma | KO:K16198 |
| 5347 | PLK1 | polo like kinase 1 | KO:K06631 |
| 7465 | WEE1 | WEE1 G2 checkpoint kinase | KO:K06632 |
| 494551 | WEE2 | WEE1 homolog 2 | KO:K06632 |
| 9088 | PKMYT1 | protein kinase, membrane associated tyrosine/threonine 1 | KO:K06633 |
| 902 | CCNH | cyclin H | KO:K06634 |
| 1022 | CDK7 | cyclin dependent kinase 7 | KO:K02202 |
| 64682 | ANAPC1 | anaphase promoting complex subunit 1 | KO:K03348 |
| 29882 | ANAPC2 | anaphase promoting complex subunit 2 | KO:K03349 |
| 996 | CDC27 | cell division cycle 27 | KO:K03350 |
| 29945 | ANAPC4 | anaphase promoting complex subunit 4 | KO:K03351 |
| 51433 | ANAPC5 | anaphase promoting complex subunit 5 | KO:K03352 |
| 8881 | CDC16 | cell division cycle 16 | KO:K03353 |
| 51434 | ANAPC7 | anaphase promoting complex subunit 7 | KO:K03354 |
| 8697 | CDC23 | cell division cycle 23 | KO:K03355 |
| 10393 | ANAPC10 | anaphase promoting complex subunit 10 | KO:K03357 |
| 51529 | ANAPC11 | anaphase promoting complex subunit 11 | KO:K03358 |
| 246184 | CDC26 | cell division cycle 26 | KO:K03359 |
| 25847 | ANAPC13 | anaphase promoting complex subunit 13 | KO:K12456 |
| 991 | CDC20 | cell division cycle 20 | KO:K03363 |
| 9232 | PTTG1 | pituitary tumor-transforming 1 | KO:K06635 |
| 10744 | PTTG2 | pituitary tumor-transforming 2 | KO:K06635 |
| 9700 | ESPL1 | extra spindle pole bodies like 1, separase | KO:K02365 |
| 8243 | SMC1A | structural maintenance of chromosomes 1A | KO:K06636 |
| 27127 | SMC1B | structural maintenance of chromosomes 1B | KO:K06636 |
| 9126 | SMC3 | structural maintenance of chromosomes 3 | KO:K06669 |
| 10735 | STAG2 | stromal antigen 2 | KO:K06671 |
| 10274 | STAG1 | stromal antigen 1 | KO:K06671 |
| 5885 | RAD21 | RAD21 cohesin complex component | KO:K06670 |
| 7272 | TTK | TTK protein kinase | KO:K08866 |
| 699 | BUB1 | BUB1 mitotic checkpoint serine/threonine kinase | KO:K02178 |
| 9184 | BUB3 | BUB3, mitotic checkpoint protein | KO:K02180 |
| 701 | BUB1B | BUB1 mitotic checkpoint serine/threonine kinase B | KO:K06637 |
| 8379 | MAD1L1 | MAD1 mitotic arrest deficient like 1 | KO:K06638 |
| 4085 | MAD2L1 | mitotic arrest deficient 2 like 1 | KO:K02537 |
| 10459 | MAD2L2 | mitotic arrest deficient 2 like 2 | KO:K13728 |
| 51343 | FZR1 | fizzy and cell division cycle 20 related 1 | KO:K03364 |
| 8555 | CDC14B | cell division cycle 14B | KO:K06639 |
| 8556 | CDC14A | cell division cycle 14A | KO:K06639 |
| 545 | ATR | ATR serine/threonine kinase | KO:K06640 |
| 472 | ATM | ATM serine/threonine kinase | KO:K04728 |
| 7157 | TP53 | tumor protein p53 | KO:K04451 |
| 1111 | CHEK1 | checkpoint kinase 1 | KO:K02216 |
| 11200 | CHEK2 | checkpoint kinase 2 | KO:K06641 |
| 1387 | CREBBP | CREB binding protein | KO:K04498 |
| 2033 | EP300 | E1A binding protein p300 | KO:K04498 |
| 5591 | PRKDC | protein kinase, DNA-activated, catalytic polypeptide | KO:K06642 |
| 4193 | MDM2 | MDM2 proto-oncogene | KO:K06643 |
| 1647 | GADD45A | growth arrest and DNA damage inducible alpha | KO:K04402 |
| 4616 | GADD45B | growth arrest and DNA damage inducible beta | KO:K04402 |
| 10912 | GADD45G | growth arrest and DNA damage inducible gamma | KO:K04402 |
| 5111 | PCNA | proliferating cell nuclear antigen | KO:K04802 |
| 2810 | SFN | stratifin | KO:K06644 |
| 993 | CDC25A | cell division cycle 25A | KO:K06645 |
| 4998 | ORC1 | origin recognition complex subunit 1 | KO:K02603 |
| 4999 | ORC2 | origin recognition complex subunit 2 | KO:K02604 |
| 23595 | ORC3 | origin recognition complex subunit 3 | KO:K02605 |
| 5000 | ORC4 | origin recognition complex subunit 4 | KO:K02606 |
| 5001 | ORC5 | origin recognition complex subunit 5 | KO:K02607 |
| 23594 | ORC6 | origin recognition complex subunit 6 | KO:K02608 |
| 4171 | MCM2 | minichromosome maintenance complex component 2 | KO:K02540 |
| 4172 | MCM3 | minichromosome maintenance complex component 3 | KO:K02541 |
| 4173 | MCM4 | minichromosome maintenance complex component 4 | KO:K02212 |
| 4174 | MCM5 | minichromosome maintenance complex component 5 | KO:K02209 |
| 4175 | MCM6 | minichromosome maintenance complex component 6 | KO:K02542 |
| 4176 | MCM7 | minichromosome maintenance complex component 7 | KO:K02210 |

Additional file 1 Table S2: Comparison of Survival Curves of each cluster by Log-rank (Mantel-Cox) test.

| Comparison of Survival Curves | Cluster 1 vs Cluster 2 | Cluster 1 vs Cluster 4 | Cluster 1 vs other Clusters |
| --- | --- | --- | --- |
| Chi square | 2.948 | 4.841 | 3.309 |
| df | 1 | 1 | 1 |
| P value | 0.086 | 0.0278 | 0.0689 |
| Significant different^†^ | ns | * | ns |

^†^ ns represents non-significance

*P-values were less than 0.05

Additional file 1 Table S3: Comparison of individual gene expressions of each cluster.

| Genes | Mean Difference^†^ | Absolute value of  Mean Difference | Significance^‡^ |
| --- | --- | --- | --- |
| CCNE1 | 21.53629374 | 21.53629374 | 6.96046E-26 |
| WEE2 | 4.878710923 | 4.878710923 | 2.89085E-09 |
| YWHAH | -1.572557852 | 1.572557852 | 1.04575E-08 |
| CDKN2A | 1.387680919 | 1.387680919 | 2.27625E-07 |
| E2F4 | -1.247110962 | 1.247110962 | 7.67536E-06 |
| TTK | 1.234002067 | 1.234002067 | 2.11172E-13 |
| SFN | -1.091020421 | 1.091020421 | 9.34955E-05 |
| TP53 | -1.082619694 | 1.082619694 | 3.72632E-05 |
| CDC25C | 1.000457325 | 1.000457325 | 5.52801E-08 |
| CDC14B | -0.997022861 | 0.997022861 | 1.79967E-05 |
| ESPL1 | 0.980717785 | 0.980717785 | 5.24243E-05 |
| CDK7 | -0.959462096 | 0.959462096 | 3.26439E-05 |
| HDAC1 | -0.736011426 | 0.736011426 | 0.001917822 |
| CCNE2 | -0.651164507 | 0.651164507 | 0.001056983 |
| MCM5 | -0.645756823 | 0.645756823 | 0.003143052 |
| MCM6 | -0.567696612 | 0.567696612 | 0.004233204 |

^†^The means of the cluster 1 minus the corresponding means of the other cases

^‡^Significance of ANOVA analysis was represented by P value

Supplementary Table 4: Correlation between Cluster-specific genes expression and tumor stages.

| Cluster-specifically expressed genes | Pearson correlation^†^ | Significance^‡^ |
| --- | --- | --- |
| *ESPL1* | -0.25713 | 9.53929E-05 |
| *FZR1* | -0.20806 | 0.001345318 |
| *CDC25C* | -0.13984 | 0.022499508 |
| *MCM5* | -0.13982 | 0.022512055 |
| *SFN* | -0.13783 | 0.024094047 |
| *CDC20* | -0.13048 | 0.030790584 |
| *GADD45B* | 0.11454 | 0.050569157 |
| *RBL2* | 0.121327 | 0.041175722 |
| *CDC14B* | 0.126105 | 0.035447174 |
| *ATM* | 0.135046 | 0.026473547 |
| *SMAD2* | 0.13647 | 0.025235082 |
| *ANAPC13* | 0.140608 | 0.021907706 |
| *ORC3* | 0.146489 | 0.017817284 |
| *ATR* | 0.165138 | 0.008844436 |
| *ANAPC4* | 0.184581 | 0.003953983 |

^†^Person correlation coefficient of gene expressions vs tumor stages

^‡^Significance of regression analysis was represented by P value
