# Supplementary figures and images for: Expression and prognostic value of cell-cycle-associated genes in gastric adenocarcinoma
Source: BMC Gastroenterol. 2018 Jun 8;18:81. doi: 10.1186/s12876-018-0811-1 (PMC5994033; doi:10.1186/s12876-018-0811-1)

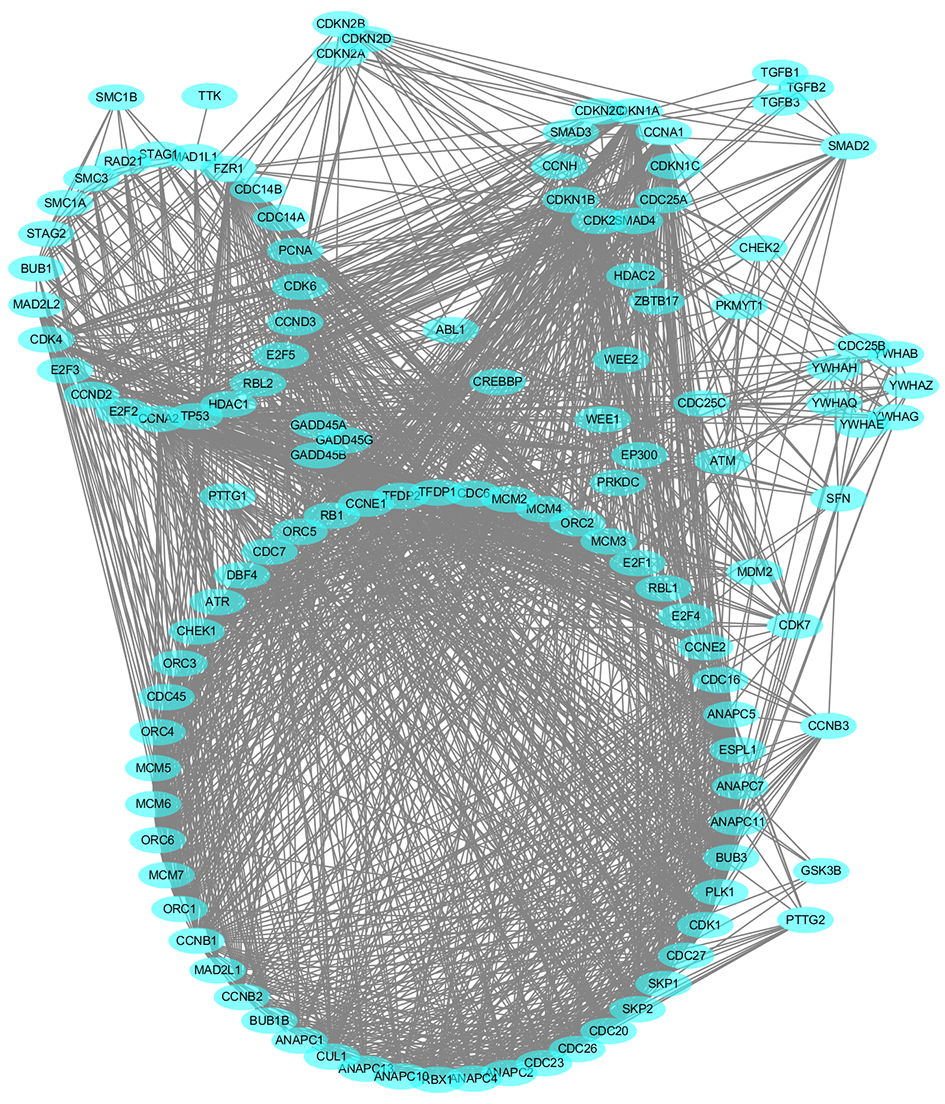

Supplement: Supplementary file 2 — Figure S1. Networks of cell cycle associated genes. The interaction values between each connected gene were exported from String-db. (TIF 1159 kb). [file 12876_2018_811_MOESM2_ESM.tif]
